# Effects of Exposure Timing on *cyp1a* Expression, PAH Elimination, and Lipid Utilization in Lumpfish Embryos Exposed to Produced Water

**DOI:** 10.1021/acs.est.2c08658

**Published:** 2023-05-12

**Authors:** Bjørn Henrik Hansen, Augustine Arukwe, Hannah Marie Knutsen, Kaja Skarpnord, Julia Farkas, Lara Veylit, Raymond Nepstad, Essa Ahsan Khan, Trond Nordtug, Lisbet Sørensen

**Affiliations:** † Climate and Environment, SINTEF Ocean, N-7465 Trondheim, Norway; ‡ Department of Biology, Norwegian University of Science and Technology, N-7491 Trondheim, Norway; § Department of Materials Science and Engineering, Norwegian University of Science and Technology, N-7491 Trondheim, Norway

**Keywords:** lumpsucker, oil, petroleum, gene expression, bioaccumulation, uptake

## Abstract

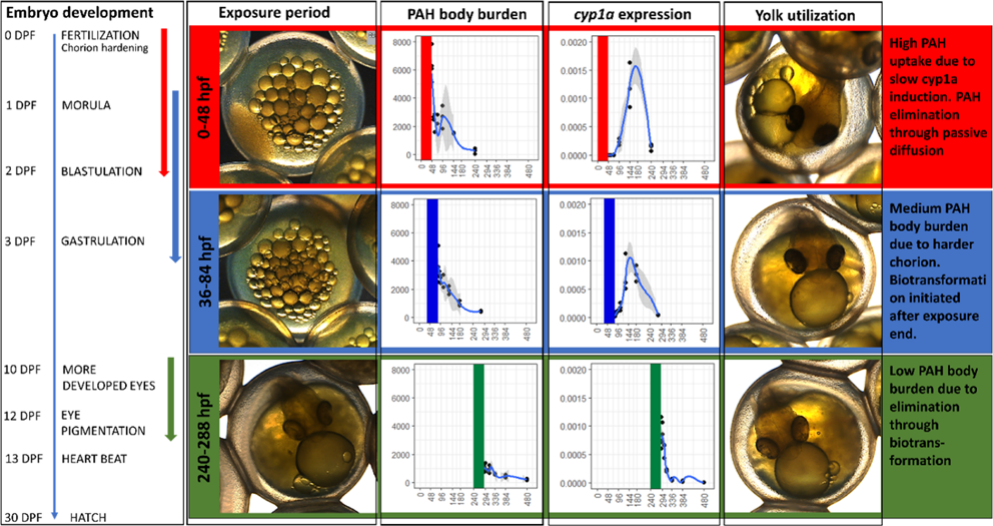

Intentional discharges of produced water from oil production platforms to the marine environment contain a complex mixture of toxicants, including polycyclic aromatic hydrocarbons (PAHs). Early life stages of fish are highly sensitive to petrogenic exposure, and short-term exposure during critical periods of embryonic development may have detrimental effects on larvae health and survival. However, why different periods are more sensitive to exposure than others are not fully understood. Three identical exposure experiments (48 h, approx. 30 μg/L tPAH, sum 42 PAHs) on lumpfish (*Cyclopterus lumpus*) embryos were conducted where only exposure timing was varied: 0–48 h post fertilization (hpf, starting before chorion hardening), 36–84 hpf (starting after chorion hardening), and 240–288 hpf (during organogenesis). Total PAH (tPAH) uptake at the end of exposure was 5× higher when exposed during fertilization than when exposed late (during organogenesis). The first evidence of *cyp1a* induction in lumpfish during embryogenesis was observed after 84 hpf. Early exposure affected lipid droplet coagulation, indicating altered lipid utilization during embryogenesis. Although no significant impacts of exposure were observed on hatching success, hatching was delayed when exposed at the latest time point. This study shows that chorion properties, lipid content, biotransformation potential, and timing of produced water exposure during lumpfish embryogenesis affected PAH uptake and elimination.

## Abstract 2

Due to differences in chorion properties and biotransformation ability, timing of fish embryo exposure determines PAH uptake and elimination. Exposure timing is critical for PAH uptake and elimination in fish embryos. Differences in PAH kinetics are associated with chorion properties and biotransformation potential. The first sign of *cyp1a* induction in lumpfish embryos was at 84 h post fertilization. Early produced water exposure causes altered lipid droplet coagulation and alterations in fatty acid profiles.

## Introduction

Intentional discharges of produced water (PW) from oil production platforms to the marine environment contain a complex mixture of potential toxicants, including polycyclic aromatic hydrocarbons (PAHs). Oil production occurs in relatively shallow water on the Norwegian Continental Shelf, and PW discharge plumes geographically overlap with spawning grounds of commercially important fish species,^1^ and, thus, there is a risk for uptake of potentially toxic PW components in developing fish embryos, leading to effects that could subsequently impact fish stock recruitment.^1−3^

Early life stages of fish are highly sensitive to petrogenic exposure, and even short-term exposure during critical periods of embryonic development can have detrimental effects on juvenile survival and population recruitment.^4−6^ Different species display varying sensitivity to exposure to chemicals,^2^ but sensitivity also varies between different periods of embryogenesis in marine fish.^7−10^ In Atlantic haddock (*Melanogrammus aeglefinus*), exposure between gastrulation and cardiac cone stage (before first heart beat) caused higher PAH uptake and more severe effects than exposure of embryos from first heart beat to one day before hatch.^10^ Similarly, in Atlantic cod (*Gadus morhua*), timing of exposure during embryogenesis affected PAH uptake, mortality rates, and timing of mortality.^9^ Intraspecies variations in PAH uptake and sensitivity may be attributed to differences in the ability to metabolize PAHs through aryl hydrocarbon receptor (*ahr*) mediated gene expression of cytochrome P450 1A (*cyp1a*).^10^ Differences in egg chorion properties may also explain the intra- and interspecific differences in sensitivity to petrogenic exposure.^5,7,10,11^ Differences in the ability for dispersed oil droplets to adhere to chorion was observed between cod and haddock^11^ and between different embryonic developmental stages within each species.^7,10^ Adhesion of oil droplets to the chorion causes increased uptake of petrogenic compounds in embryos,^12^ and the presence of oil droplets during exposure of pelagic fish eggs to oil dispersions causes higher toxicity than when exposed to filtered dispersion not containing droplets, but comparable composition of dissolved PAHs.^7,9,10,13^

In the presence of activating spermatozoa and seawater, fertilized marine fish eggs undergo a calcium-driven cortical reaction increasing their osmolarity and thickness and surface appearance, resulting in increased chorion strength, resistance, and hardness.^14,15^ Morphological features, like chorion appearance and strength, vary between species.^16^ Due to their hydrophobic properties, PAHs are known to affect membrane integrity and ion regulation^17^ and may therefore affect the chorion hardening process, and its permeability. Intriguingly, no data exist on the effect of the chorion hardening process on the uptake of PAHs in fish embryos.

Lumpfish (*Cyclopterus lumpus*) is a semi-pelagic fish species, widely distributed throughout the North Atlantic Ocean and the Barents Sea,^18^ and although they usually spawn in the sub-littoral zone in coastal areas, populations of lumpfish also spawn and attach their fertilized eggs to the legs of North Sea oil production platforms.^19^ These demersal eggs have a thick and complex chorion, and they are lipid rich with heterogenous yolk consisting of both yolk and lipid droplets. They develop relatively slowly (approximately 300 day-degrees, dd) but hatch at a more advanced stage than pelagic eggs.^20^

Given the demonstrated sensitivity of early life stages of fish to crude oil pollution, lumpfish embryos developing close to PW discharges are likely to be at risk of accumulating PW compounds. Lumpfish is regarded as a robust species, with an ability to tolerate a wide range of temperature and oxygen tension^21^ and with juveniles being relatively resilient to experimental oil exposures.^22^ The chorion of newly fertilized lumpfish eggs is considered sticky until hardening and characterized as “being glasslike” thereafter, potentially preventing the transfer of water-soluble contaminants across membranes. Fertilized lumpfish eggs are considerably harder than, e.g., cod eggs, displaying a resistance of 2000 g compared to cod, which has a resistance of only 150 g.^15^ However, lumpfish eggs are lipid-rich and develop over a long time (29–30 days at 10 °C)^21,23^ and thus have a high potential for accumulation of lipophilic contaminants. The properties of fertilized lumpfish eggs are beneficial for in situ deployment to assess the exposure and toxicity of chemical stressors in the marine environment.^2^ However, no information is available about the ability of lumpfish embryos to accumulate and metabolize lipophilic contaminants like PAHs.

The aim of the current study was to investigate the potential for accumulation and elimination of PW-derived PAHs in embryos of lumpfish, and importantly, the effect of exposure timing during embryonic development. Increased knowledge about these processes is important for environmental risk assessment of PW discharges, is vital when using lumpfish embryos for environmental monitoring, and can be used to understand sensitivity differences between fish species in relation to PW exposure.

## Experimental Section

### Chemicals and Materials

Certified standard solutions of PAHs, alkylated PAHs, heteroaromatics, and deuterated PAHs were purchased from Chiron AS (Trondheim, Norway). All solvents were of analytical grade, and purity was verified in-house before use. PW was collected and acidified (HCl, pH < 2) at the point of release of a Norwegian Sea offshore oil-producing platform. Upon arrival at the onshore laboratory, the PW (195 L) was solvent extracted using dichloromethane, as described previously,^25^ and concentrated to 250 mL. Extracts were stored dark and frozen (−20 °C) until further use in experiments.

### Preparation of Exposure Solutions

Stock solutions for exposure were prepared by re-constitution of the PW extract in seawater as described previously.^2,25^ The nominal stock concentration aimed for was 100 μg/L of the total PAH (sum of 42 PAH; tPAH). Briefly, 7 mL of the total PW extract was transferred to a 2 L glass bottle, the solvent was evaporated at 40 °C under a gentle stream of N_2_, 1.4 L sterile filtered (0.22 μm Sterivex cartridges) seawater was added, and the bottles were ultrasonicated. To achieve the experimental concentration (nominally 30 μg/L tPAH), the stock solution was diluted with additional sterile seawater that had been aerated under sterile conditions. The solutions were acclimated overnight to the experimental temperature (10 ± 1 °C).

### Animal Husbandry and Exposure Regimes

Unfertilized eggs of lumpfish were kindly donated by MOVI (Rissa, Norway) and transferred to the laboratory in a cooling container. Cryopreserved milt was provided by Cryogenetics (Hamar, Norway) and delivered frozen in liquid nitrogen. Eggs were fertilized by mixing 45 mL eggs, 25 mL seawater (or exposure solution), and 250 μL milt in a glass beaker. The beaker with content was incubated for approximately 2 min before the fertilized eggs were transferred to molds prepared of plastic plates with circular holes (diameter: 2.5 cm). Fertilized eggs (80–90 eggs) were gently placed as monolayers inside the holes. Seawater (or exposure solution) was gently poured over to cover the eggs, and the eggs were kept for hardening for 30 min. After hardening, the fertilized eggs were either transferred directly into the exposure solution (100 mL) in glass beakers or into flow-through incubator tubes made from 50 mL conical polypropylene (PP) centrifuge tubes (Falcon, Corning Life Sciences) modified to allow flow-through. Each tube was cut, a Teflon mesh (mask width: 300 μm) was molded to the lower part, and a replaceable mesh (mask width: 300 μm) was placed below a screw cap with the central part removed (Figure S1A). The incubator tubes were fitted into the holes of a horizontal plate (polycarbonate) separating the upper and lower volumes of a plastic box. Filtered seawater (1 μm filtered, 10 °C) was supplied to the bottom section of the plastic box containing the inserted incubator tubes (Figure S1B) at a rate of 4 L/min and drained from the upper volume after passing through each tube at an average rate of 35 mL/min.

Fertilized lumpfish eggs were subjected to 48 h exposure at three different time points; 0–48 h post fertilization (hpf), 36–84 hpf, and 240–288 hpf [10–12 days post fertilization (dpf)], hereafter called DEP1, DEP2, and DEP3, respectively. Representative images of embryos at the end of exposure are given in the Supporting Information (Figure S2). DEP1 covers the period from fertilization, including the hardening process, to blastulation; DEP2 starts at blastulation and continues into the onset of gastrulation; and DEP3 covers the period where eyes are getting pigmented to heart beats become visible.^26^

Exposure media was renewed every 24 h. After exposure, the samples were transferred to clean, running seawater in a custom-made incubator system^24^ and kept until hatch. Subsamples of lumpfish embryos were taken for body burden, lipid content, and gene expression analyses at seven time points [0, 6, 12, 24, 48, 96, and 192 h post exposure (hpe)]. Samples were preserved by flash-freezing in liquid nitrogen and stored at −80 °C until further handling. For all exposures, *N* = 6, and for controls *N* = 4. Images were taken of embryos and larvae at several time points during the experiment to visually inspect development during embryogenesis. Images of larvae were used to determine the area of larvae body and the fraction of the body area covered by the yolk sac and lipid droplets using AUTOMOMI,^27^ and lipid coagulation was observed visually on images.

### Fertilization, Hatching, and Survival

Fertilization rate was recorded a week after fertilization, and eggs with no clear developing embryo were defined as non-fertilized. Embryos that were not hatched by 32 dpf were visually inspected for heart activity under the microscope and considered dead if no heartbeat was observed. Hatching was monitored daily, and hatching success and timing was recorded as the number of embryos hatching with viable larvae between 28 and 32 dpf.

### RNA Extraction and *cyp1a* Gene Expression Analyses

Total RNA was isolated from 30–40 fertilized eggs using the Direct-zol RNA MiniPrep kit following the manufacturer’s procedures. Complementary DNA (cDNA) was synthesized from 1 μg total RNA using the iScript cDNA synthesis kit in accordance with the included protocol (Bio-Rad, Oslo, Norway). RNA isolation, cDNA synthesis, and real-time PCR analysis was performed based on the standard protocol and as described previously.^28,29^ The following primer pairs were used for lumpfish *cyp1a* (accession number: XM_034540817): Forward primer: GATGTACTTGGTGGCTTACC. Reverse primer: GAAGGAGCTCAAGGATGAAG (product size of 143 bp).

### Exposure Characterization

Samples of the exposure media (∼200 mL) were taken before and after (pooled sample from all replicates) renewal in the exposure beakers. Samples were acidified (HCl, pH < 2) and stored dark and cool (4 °C) until further handling. Surrogate internal standards (250.8 ng naphthalene-*d*_8_, 50.0 ng phenanthrene-*d*_10_, 48.6 ng chrysene-*d*_12_, and 50.8 ng perylene-*d*_12_) were added prior to extraction to account for analyte loss during extraction. The samples were extracted three times by partitioning to dichloromethane and dried with Na_2_SO_4_. The sample volume was adjusted by gentle evaporation, and a recovery internal standard (98.4 ng fluorene-*d*_10_) was added. An Agilent 7890B with an Agilent 5977A quadrupole MS fitted with an EI source was used for analysis of the water samples. A DB5 MS UI column (60 m × 0.25 mm × 0.25 μm) was used for separation. The carrier gas was helium, at a constant flow rate of 1 mL/min. Samples (1 μL) were injected in pulsed splitless mode at 325 °C. The oven was held at 40 °C (1.4 min) and ramped by 6 °C/min to 220 °C and by 40 °C/min to 325 °C (held for 10 min). The transfer line temperature was 325 °C. The MS was operated at 70 eV in selected ion monitoring (SIM) mode with the ion source at 230 °C and the quadrupole at 150 °C. The analytes were identified by their molecular and fragment ions ion. Quantification was based on average response factors relative to internal standard fluorene-*d*_10_. The following 42 PAHs were included in the analyses of water samples and body burden: C_0_–C_4_-naphthalenes, biphenyl, acenaphthylene, acenaphthene, dibenzofuran, C_0_–C_3_-fluorenes, C_0_–C_4_-phenanthrenes/anthracenes, C_0_–C_4_-dibenzothiophenes, C_0_–C_3_-fluoranthenes/pyrenes, benz(*a*)anthracene, C_0_–C_3_-chrysenes, benzo(*b*)fluoranthene, benzo(*k*)fluoranthene, benzo(*e*)pyrene, benzo(*a*)pyrene, perylene, indeno(*1*,*2*,*3-c*,*d*)pyrene, dibenz(*a*,*h*)anthracene, and benzo(*g*,*h*,*i*)perylene. The summed concentrations of these PAHs are referred to as total PAH (tPAH).

### PAH Body Burden Analyses

Extraction of tissue samples was performed as described by Sørensen et al.,^30,31^ with minor modifications. After addition of surrogate standards (25.08 ng naphthalene-*d*_8_, 5.0 ng phenanthrene-*d*_10_, 4.86 ng chrysene-*d*_12_, and 5.08 ng perylene-*d*_12_), pooled samples (∼25 eggs) were homogenized in *n*-hexane–dichloromethane (DCM) (1:1 v/v, 4 mL) using a glass rod, followed by the addition of Na_2_SO_4_, vortex mixing, and centrifugation. The supernatant was collected, and the extraction was repeated twice. The combined organic extract was concentrated to approximately 1 mL prior to clean-up by silica solid phase extraction (SPE, Supelco SiOH 500 mg columns). The analyte fraction was eluted using *n*-hexane–DCM (9:1 v/v, 6 mL), followed by gentle solvent evaporation and addition of recovery internal standard (9.84 ng fluorene-*d*_10_). An Agilent 7890 GC with an Agilent 7010B triple quadrupole MS fitted with an EI source and collision cell was used for the analysis of body burden samples. Two DB-5MS UI columns (30 m × 0.25 mm × 0.25 μm) were coupled in series through a purged ultimate union (PUU). The carrier gas was helium at a constant flow rate of 1.2 mL/min. Samples (1 μL) were injected in pulsed splitless mode at 310 °C. The oven was held at 40 °C for 1.5 min, ramped to 110 °C by 40 °C/min, ramped to 220 °C by 6 °C/min, and finally ramped to 325 °C at 4 °C/min (5 min hold). The temperature was held at 330 °C for 5 min, while the first column was backflushed. The transfer line temperature was 300 °C. The MS was operated at 70 eV in multiple reaction monitoring (MRM) mode with the ion source at 230 °C and the quadrupole temperatures at 150 °C. Nitrogen was used as a collision gas (1.5 mL/min), and helium was used as a quench gas (2.25 mL/min). The analytes were identified by two unique MRM transitions (Sørensen et al., 2016). Quantification was performed by quadratic regression (parent PAHs) or average response factors (alkyl PAHs) relative to internal standard fluorene-*d*_10_.

### Lipid Content and Composition

Total lipid content was determined in pooled samples (∼10 fertilized eggs) following a modified Folch extraction.^32^ Samples were homogenized in chloroform–methanol (2:1 v/v, 4 mL), centrifuged (2000 rpm, 10 min), and the supernatant was collected. NaCl (0.9% in MilliQ-water, 1 mL) was added, and the sample was centrifuged (2000 rpm, 5 min). The organic phase was isolated and evaporated to dryness. The weight of the total extracted lipid was recorded.

Fatty acid composition was determined by fatty acid transmethylation to fatty acid methyl esters (FAMEs) and GC-FID analysis. Lipid extracts were dissolved in 1 mL 0.1 M NaOH in methanol and heated to 100 °C for 15 min. Next, 2 mL 50% boron trifluoride in methanol were added, and the reactions were heated to 100 °C for 5 min. The reactions were allowed to cool to room temperature, and 1 mL hexane was added before heating to 100 °C for 1 min. FAMEs were extracted by adding 1 mL hexane and 2 mL of a saturated NaCl solution. The phases were separated by centrifugation at 2000 rpm for 5 min, and the upper phase was collected in a new tube. The extraction was repeated twice with 1 and 2 mL hexane and mixed. The hexane-extracted FAMEs were finally subjected to analysis by GC-FID. The FAMEs were analyzed according to Daukšas et al.^33^ with the following modifications: an Agilent Technologies 7890A gas chromatograph with flame ionization detection (GC-FID) equipped with a 7693 autosampler was used. The detector temperature was held at 270 °C, and the flame was maintained with 25 mL/min H_2_ gas and 400 mL/min filtered air. Chromatography was carried out using a Cp-wax 52CB, 25 m, 0.25 mm with i.d. 0.2 mm column (Agilent Technologies). Helium was used as the carrier gas at a flow rate of 1.5 mL/min. GC inlets were held at 250 °C. The initial oven temperature was held at 80 °C and increased to 180 °C at 25 °C/min, followed by a 2 min hold, after which the temperature was increased to 205 °C at 2.5 °C/min, followed by a 6 min hold, after which the temperature was increased to 215 °C at 2.5 °C/min, followed by a 4 min final hold. Fatty acids were characterized by comparison to the retention times of commercial standards and quantified by an internal standard. The accuracy of the method was verified by comparison of FA profiles of selected marine oils against profiles assessed by accredited laboratories.

### Data Treatment and Statistical Analyses

To describe how the *cyp1a* expression and tPAH body burden changed over time (i.e., hours post exposure), we fitted local regressions. A non-parametric approach was applied due to small sample sizes across the exposure groups. Curve fit was used for visual assessments of the trends in the data. Models fit to *cyp1a* expression and tPAH body burden were fit in R (v. 4.1.2) using the package ggplot2 (v. 3.3.6).^34^ Elimination rates for each PAH were estimated using a scaled internal concentration model, which is a simple exponential decay model . This was done by minimizing the negative log likelihood function obtaining the best single value of *k*_e_ across replicates for each PAH. The confidence intervals around the estimates were determined by profiling the likelihood function.^35^ An inverse relationship would be expected between *k*_e_ and *K*_ow_ for PAHs, and different functional relationships have been proposed.^36,37^ The OMEGA model was used to determine *k*_e_ from a small set of chemical and biological descriptors such as *K*_ow_, lipid fraction, and weight.^38^ Here, we fitted a log-linear relationship between *k*_e_ and *K*_ow_ for each DEP experiment using the estimated elimination rates and their calculated confidence interval
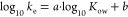


These fits and the estimation of the elimination rates were performed with Python 3.8, using the lmfit package version 1.0.2.^39^ Statistical analyses comparing data on the obtained variables for different treatments were conducted using One-way ANOVA followed by Dunnett’s multiple comparisons test using GraphPad Prism version 9.4.1 for Windows (GraphPad Software, San Diego, California USA).

## Results and Discussion

### Exposure Profiles

**Figure 1:**
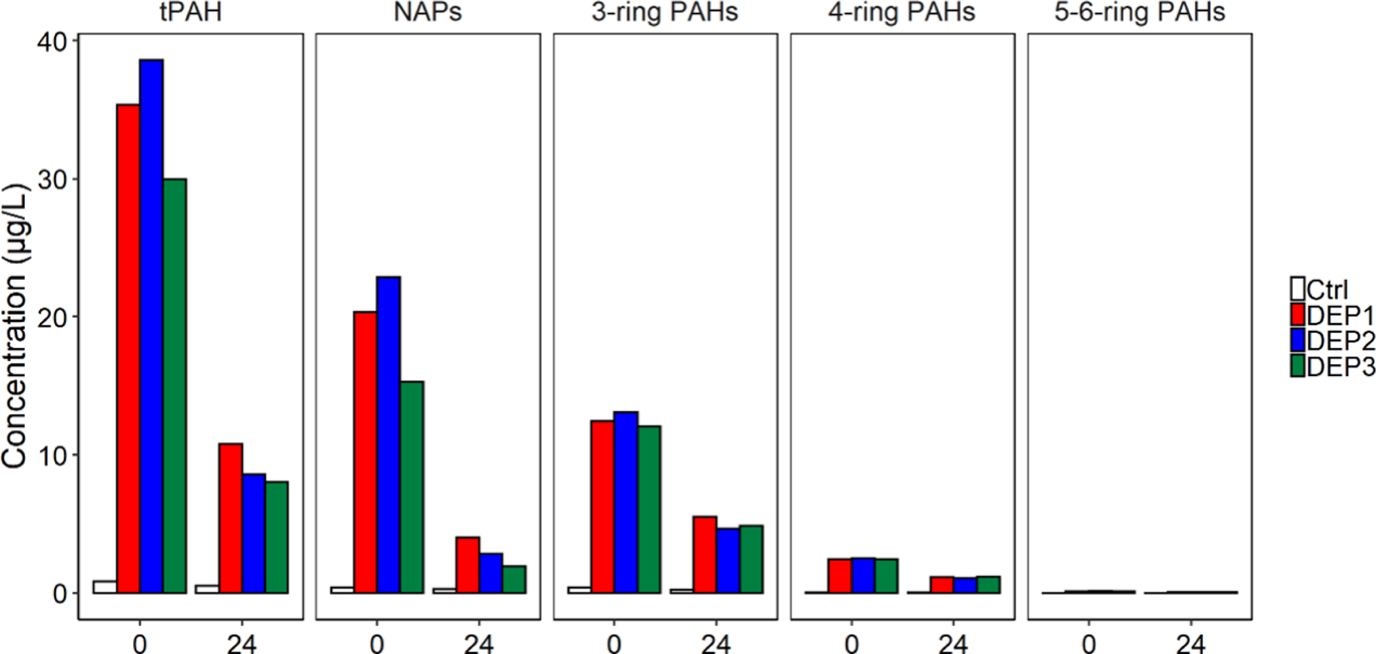
Concentrations (μg/L) of total (t)PAH, C_0_–C_4_-naphthalenes (NAPs), 3-ring and 4-ring PAHs (including alkylated homologues), and 5- to 6-ring PAHs in exposure solutions at the start (0) and after 24 h of exposure at three different time points (DEP1 is red, DEP2 is blue, and DEP3 is green) compared to control exposure samples (white).

PW is a complex mixture of thousands of different chemical components; thus, multiple components may therefore represent risk-driving components for environmental impacts of PW.^1^ Petrogenic compounds are a large fraction of PW, and the concentration of PAHs appears to correlate to the toxicity in developing fish.^1,10,40−42^ The aim of the current study was to investigate the accumulation and biotransformation of PW-derived PAHs in lumpfish embryos exposed at different ages. Lumpfish oocytes were randomly distributed to four treatment groups (one control group and three exposure groups). The exposure groups were exposed to re-constituted PW (PWR) at different time points [DEP1 = 0–48 h post fertilization (hpf), DEP2 = 36–84 hpf, and DEP3 = 10–12 dpf]. Exposure solutions were made fresh for each exposure, aiming for a nominal tPAH concentration of approximately 30 μg/L. The concentrations of PAHs measured in exposure solutions before use and after 24 h in beaker exposures is shown in Figure 1.

The measured exposure concentrations at exposure start were 30–40 μg tPAH/L, and the solutions were renewed at 24 h. The concentration of naphthalenes in the prepared exposure solutions for DEP3 were slightly lower than for DEP1-2, likely due to volatility losses, but concentrations of 3- to 6-ring PAHs were comparable in all three experiments. A significant loss of PAHs from the exposure solutions was observed after 24 h, and as such, this semi-static experimental approach can be considered a pulsed exposure. The loss could be attributed to a combination of loss due to uptake and metabolization in embryos, due to evaporation from the open beakers (mainly naphthalenes), and due to glass beaker wall sorption (mainly affecting larger compounds). The tPAH concentrations in these lumpfish experiments were in the range where toxicity to early life stages of marine fish have previously been reported. In cod and haddock, exposure of embryos to reconstituted PW with tPAH concentrations of approximately 10 μg/L caused severe craniofacial, jaw, and spine deformations.^2^

### PAH Accumulation in Fish at Different Developmental Stages

**Figure 2:**
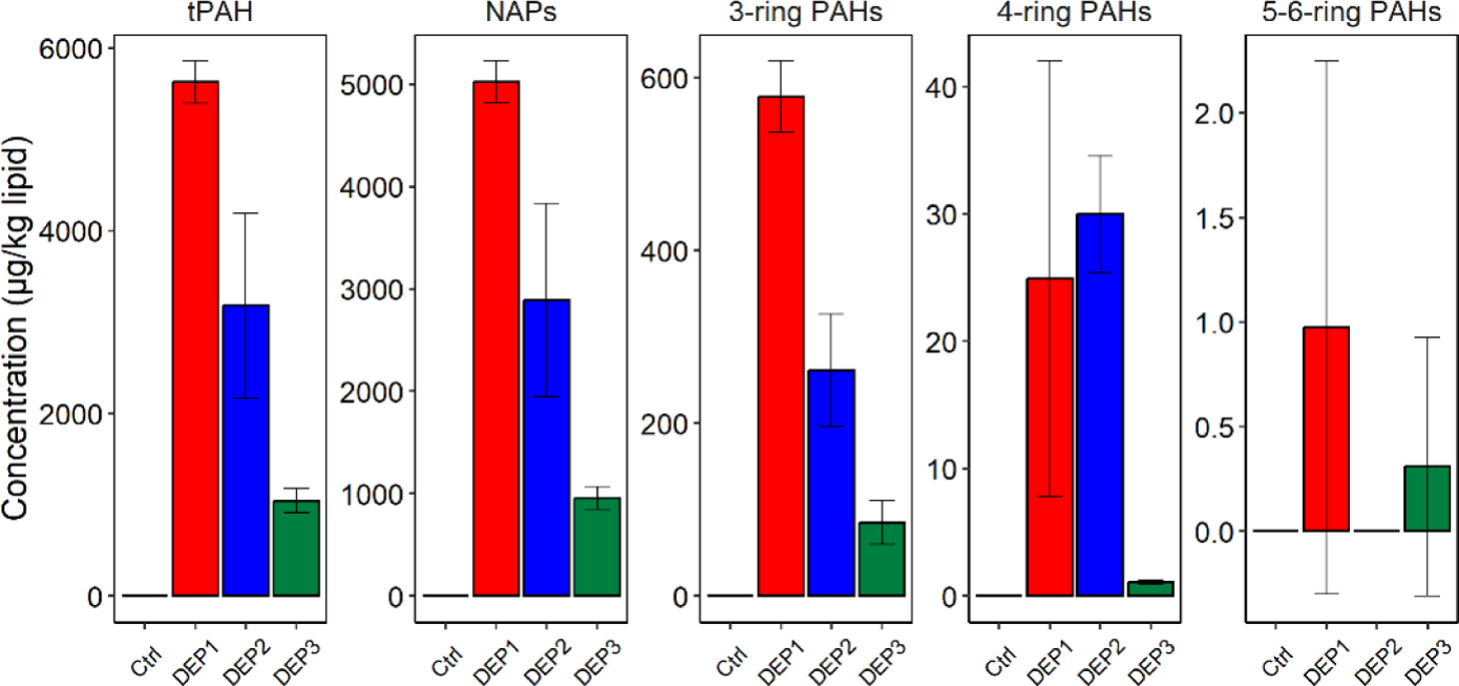
Lipid-normalized PAH concentrations in fertilized lumpfish eggs after 48 h exposure. DEP1: exposed 0–48 hpf, DEP2: exposed 36–84 hpf, and DEP3: exposed 10–12 dpf. Controls were exposed to filtered seawater only. Error bars show standard deviation (*n* = 3). Please note the difference in the *y*-axis scale between the panels.

Sensitivity to petrogenic exposure varies between different periods of embryogenesis in marine fish,^7−10^ and this may be linked to toxicokinetic differences. After 48 h exposure, the highest tPAH body burdens were found in DEP1 (exposed 0–48 hpf), the lowest were found in DEP3 (exposed 10–12 dpf), and DEP2 was intermediate (Figure 2). Thus, exposure timing was critical for PAH accumulation in lumpfish embryos, with exposure before and during the egg hardening process (DEP1) causing higher PAH uptake than exposure after hardening (DEP2). The even lower PAH body burden at the end of exposure in the DEP3 experiment can be explained by the increased ability of the embryos to metabolize PAHs at this age (see below). Similar results have previously been shown for Atlantic cod and haddock exposed to crude oil dispersions. In cod, higher PAH body burdens were observed when exposed 3–7 dpf compared to exposure at 9–13 dpf.^9^ Comparably, haddock eggs exposed 2.5–6.5 dpf displayed higher PAH body burden than eggs exposed 7.5–11.5 dpf.^10^ Importantly, previous studies have shown the capability of fish embryos to actively metabolize and eliminate PAHs even during constant exposure conditions.^5^

Contrary to the exposure media, the lumpfish embryo body burden was heavily dominated by lower-molecular-weight aromatics (naphthalenes). The naphthalene body burden was an order of magnitude higher than 3-ring PAH body burden, which in turn was an order of magnitude higher than 4-ring PAH body burden. Larger PAHs (5–6 rings) were only detectable at trace levels (Figure 2), which was unsurprising given the low concentrations in the PW exposure media and the short exposure time (48 h).

### *cyp1a* Expression and PAH Elimination

**Figure 3:**
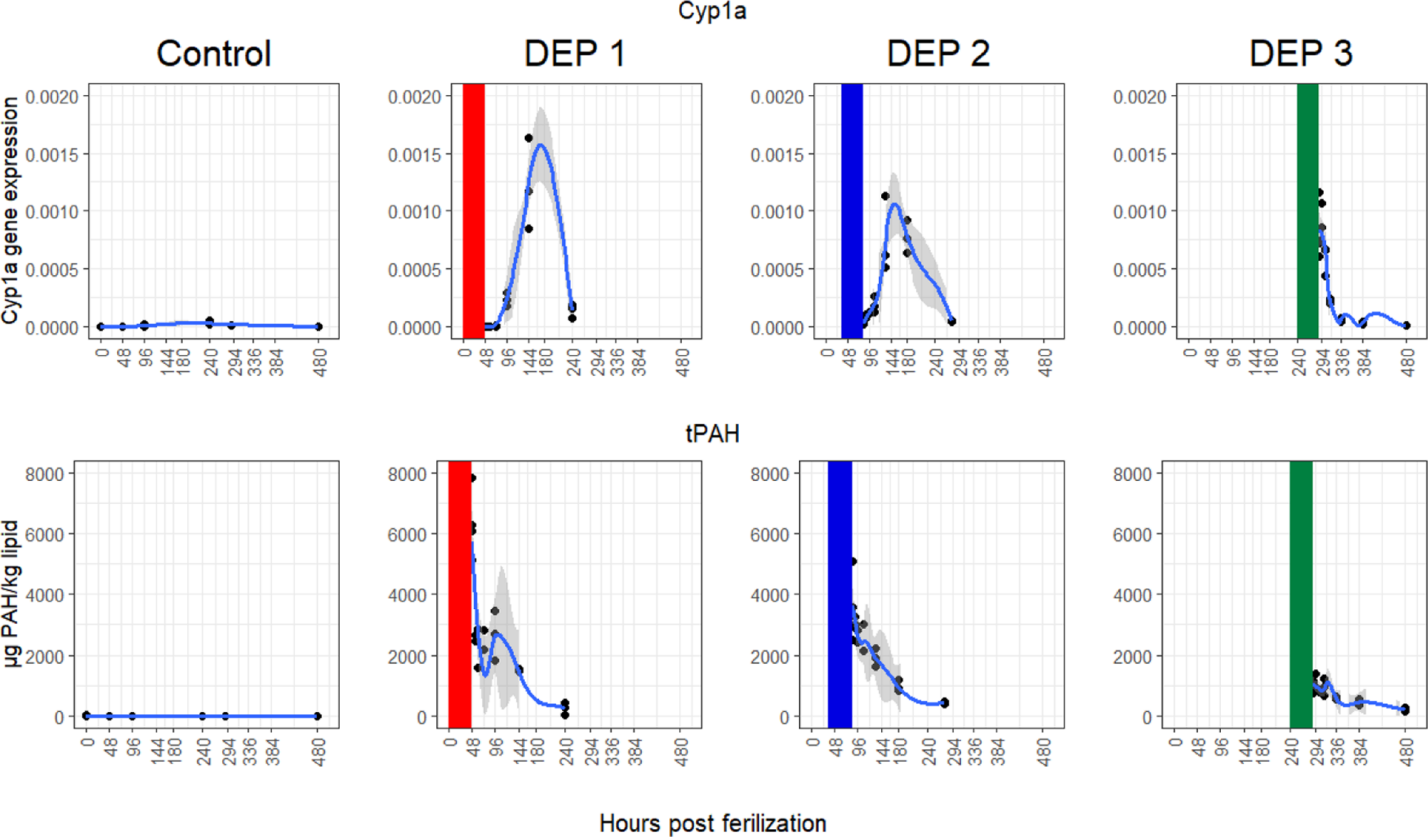
Local regressions and associated standard error fit to *cyp1a* expression (top plots) and total PAH body burden (bottom plots) data. Gene expression and body burden is shown over time (hours post fertilization) with models fit to exposure groups (i.e., DEP1, DEP2, DEP3, and control). The period during which embryos were exposed is shown with rectangles colored according to the exposure group.

Elimination of PAHs in fish occurs through two main processes: passive diffusion and active excretion. Passive diffusion from biota to the water occurs under non-equilibrium conditions, whereas active excretion requires PAH metabolism. The latter process is divided into three phases: activation of the aryl hydrocarbon receptor (*ahr*) by a substrate (e.g. PAH) to induce enzymes (like *cyp1a*) that initiates PAH oxidation (phase 1), followed by enzymes (like glutathione *S*-transferase) facilitating conjugation (phase 2),^43^ and finally, transport and excretion (phase 3).^10^*Cyp1a* gene expression and PAH body burdens were measured in lumpfish embryos at the end of exposures and 6, 12, 24, 48, 96, and 192 h after exposure for all three treatments (DEP1–DEP3) and controls. Local regressions were fit to both *cyp1a* expression and tPAH body burden data for each exposure group separately (Figure 3). For DEP1 (exposed 0–48 hpf), *cyp1a* expression was not affected until 48 h after end of exposure at the age of 96 hpf, and the highest expression was observed at 96 h post exposure (144 hpf). For DEP2 (exposed 36–84 hpf), a minor increase in the *cyp1a* expression can be observed at the age of 84 hpf, but here the highest expression was at 132–180 hpf (48–96 h post exposure). For DEP3, which were exposed at a much later stage (10–12 dpf), the *cyp1a* expression was the highest 0–6 h (288–294 hpf) after exposure, decreasing rapidly thereafter. Most likely, *cyp1a* transcription was initiated already during exposure, but this was not measured.

The earliest expression of *cyp1a* was observed for DEP2 at 84 hpf (Figure 3). Thus, before the lumpfish reached this age, PAH elimination from lumpfish embryos was driven by passive diffusion. This work represents the first study of *cyp1a* expression in lumpfish, but expression of this gene has been studied in other cold-water species subjected to petrogenic exposure.^5,5,10,29^ In Atlantic haddock, exposure to crude oil during early embryogenesis [2.5–5 dpf (10% epiboly to 10–20 somite/cardiac cone stage)], resulted in higher PAH uptake due to lower metabolism resulting in more severe abnormalities.^10^ This is in line with our observations, where lack of *cyp1a* expression at the end of exposure in the DEP1 and DEP2 experiments was accompanied by high tPAH body burdens compared to DEP3 where higher *cyp1a* expression was accompanied by much lower PAH body burdens. In haddock, late embryogenesis exposure, i.e., after first heartbeat and until the 30–40 somite stage to the hatching gland stage (7.5–10.5 dpf), a lower PAH uptake was observed possibly due to higher *cyp1a* expression.^10^ Both haddock and cod develop much faster (hatching around 100 dd) than lumpfish (300 dd), and developmental stages are distributed thereafter, e.g., gastrulation is initiated in cod at approximately 16 dd (56 hpf at 7 °C),^44^ whereas in lumpfish, gastrulation is initiated at 29.2 dd.^26^

Reduction in PAH body burden was observed over time after transfer to clean sea water for all three treatments (Figure 3). As mentioned above, PAH elimination is a function of both passive diffusion and active elimination. For organisms with limited biotransformation activity, like copepods, a negative relationship has been shown when plotting individual PAHs elimination rates (log *k*_e_) against their partitioning coefficients (log *K*_ow_).^37^ A similar trend was observed for lumpfish; however, the slope was steeper for DEP1 (−0.25) and DEP2 (−0.21) than for DEP3 (−0.09) (plots shown in the Supporting Information, Figure S3). This suggests that in early embryonic development (DEP1 and DEP2), elimination is (initially) driven by the passive process as active elimination depends on the *cyp1a* expression, and the *cyp1a* expression was only observed after the end of exposure. In contrast, for DEP3, passive and active elimination processes worked in combination as *cyp1a* was highly expressed already at the end of exposure. In line with our studies, biotransformation influenced body burden concentrations of the PAHs fluoranthene and benz(*a*)anthracene in zebrafish embryos, indicated by the presence of PAH metabolites.^45^ Analyses and detection of PAH metabolites in our studies would enable us to distinguish between biotransformed and actually eliminated PAHs and should be considered in future studies.

### Fertilization, Hatching, and Survival

Despite treatment-specific differences in PAH uptake and biotransformation dynamics, no differences in fertilization and hatching success were observed (Table S1). Fertilization success was high (>90%) for all treatments with no significant difference between treatments, suggesting that the exposure during fertilization did not reduce viability of oocytes and milt. High fertilization success after using oocytes from wild-caught lumpfish and cryopreserved milt, as done in this experiment, have previously been used successfully.^24,46^ Comparable to our results, between 1.6 and 13.8% unfertilized eggs have been reported using an identical fertilization protocol utilizing eggs from wild-caught females and cryopreserved milt.^24^ The use of eggs from broodstock females can provide varying fertilization success, which is related to the egg maturity and quality.^46^ Fertilized lumpfish eggs develop over a period of up to 300 dd; therefore, embryo development and, thus, hatch timing are affected by temperature, i.e., at lower temperatures, hatching will be delayed.^26^ Hatching success was high for all treatments (>80%), comparable to literature values,^24,26,46,47^ with no significant difference between treatments (Table S1). However, there were some variations in hatch timing. For controls, all hatched on days 28–29 post fertilization (>98.5% on 29 dpf), whereas DEP1, DEP2, and DEP3 hatched on days 29–30, 30–31, and 31–32, respectively. In our experiment, the temperature was identical in all incubators, so the delay for DEP3 cannot be attributed to differences in temperature. DEP3 was the group exposed at the latest time point, so exposure late in development caused a delay in hatch timing. Compared to Atlantic cod embryos, lumpfish embryos are more tolerant to PW exposure. Atlantic cod, exposed for 4 days to a comparable PAH composition and concentration caused mortality and severely deformed larvae. In a comparative study using lumpfish, capelin (*Mallotus villosus*), plaice (*Pleuronectes platessa*), flounder (*Platichthys flesus*), long rough dab (*Hippoglossoides platessoides*), and Atlantic cod, lumpfish was the least sensitive to dimethylnaphthalene exposure, but the difference between species was less for larvae.^48^ Others have also reported low sensitivity of lumpfish early life stages and juveniles to crude oil exposure.^22,49^

### Total Lipids and Fatty Acid Composition

**Figure 4:**
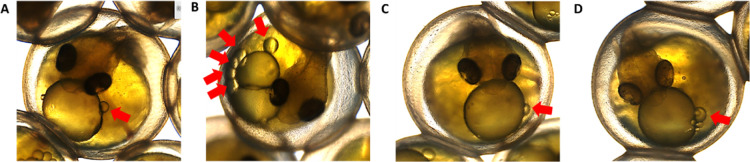
Lumpfish embryos (13 dpf) from control group (A), exposed to produced water extract 0–48 hpf (B, DEP1), 36–84 hpf (B, DEP2), and 10–12 dpf (D, DEP3).

Lumpfish eggs contain both a yolk sac and lipid droplets,^20^ and the lipid droplets coagulate into a single droplet by the age of 117 dd.^26^ In our experiment, images taken of eggs at 17 dpf (approx. 162 dd), embryos exposed at the earliest time point (DEP1) displayed dispersed lipid droplets within the eggs (Figure 4).

This was unique to the DEP1 group, which was exposed during fertilization and displayed the highest PAH accumulation after exposure. This might have been caused by the accumulation of PW components before egg hardening, which affected lipid membranes (possibly acting as surfactants) within the egg, preventing lipid droplet coagulation. At hatch, however, the number of lipid droplets and total lipid content (Figure S4) were comparable in larvae between treatments, but larvae from DEP1 and DEP2 still displayed a larger lipid area than the other groups (*p* < 0.05) (Figure S5A), suggesting that they have utilized less of the lipid droplets during development. The newly hatched larvae were also somewhat heavier in DEP1 (4.1 ± 0.1 mg) and DEP2 (4.1 ± 0.2 mg) compared to DEP3 (3.8 ± 0.2) and controls (3.8 ± 0.2 mg) (Figure S6), suggesting lack of conversion of lipids into structure. DEP1 had comparable ventral yolk sac area to the other treatments, but the yolk area was larger than controls in DEP2 and DEP3 (Figure S5B). To investigate further impacts of exposure on lipids, fatty acid (FAME) compositions were analyzed in larvae from all groups sampled 1 dph. Monosaturated fatty acids were lower in DEP1 and DEP3 compared to controls and DEP2 (*p* < 0.05), but for polysaturated fatty acids, the differences were inversed, i.e., DEP1 and DEP3 displayed higher percentage than controls and DEP2 (*p* < 0.05). These differences were mainly driven by C18:1n7 (being lower in DEP3 compared to the other groups, *p* > 0.05) and docosahexaenoic acid C22:6n3 (higher in DEP1 and DEP3 than DEP2 and control) (see Table S2 for more details). The relative composition of the unassigned peaks was also significantly lower (*p* < 0.05) for DEP1 compared to the other treatments.

Current knowledge on the process of utilizing the heterogenous yolk during embryogenesis in lumpfish is scarce,^46,50,51^ and no literature exists on the impacts of pollutants on these vital processes in lumpfish development. Previous studies have shown that suboptimal utilization of yolk in early life stages of polar cod (*Boreogadus saida*) can cause reduced long-term survival,^52^ and the impacts of embryonic PW exposure on embryonic lipid utilization and fatty acid composition shown in our study warrants more detailed studies on the underlying mechanisms and long-term effects of these impacts.

## Supporting Information Available

A schematic drawing and image of the flow-through system used for recovery of eggs post exposure, representative images of embryos taken at the end of the exposure periods, plots of *k*_e_ as a function of *K*_ow_ for all PAHs and treatments, plots of different morphometrical data on lumpfish larvae (1 dph) after extended recovery period in clean sea water, data on fertilization success, hatching success, and hatch timing, and complete information regarding FAME composition (PDF)
