# Supplementary material for: Effects of Exposure Timing on cyp1a Expression, PAH Elimination, and Lipid Utilization in Lumpfish Embryos Exposed to Produced Water
Source: Environ Sci Technol. 2023 May 12;57(20):7666–74. doi: 10.1021/acs.est.2c08658 (PMC10210533; doi:10.1021/acs.est.2c08658)
Supplement: Supplementary file 1 — es2c08658_si_001.pdf [file es2c08658_si_001.pdf]

SUPPORTING INFORMATION

Effects of exposure timing on cyp1a expression, PAH elimination and lipid utilization in lumpfish embryos exposed to produced water

Bjørn Henrik Hansen[a]*, Augustine Arukwe[b], Hannah Marie Knutsen[c], Kaja Skarpnord[c], Julia Farkas[a], Lara Veylit[a], Raymond Nepstad[a], Essa Khan[b], Trond Nordtug[a] and Lisbet Sørensen[a]

[a]*SINTEF Ocean, Climate and Environment, N-7465, Trondheim, Norway.*

[b]*Department of Biology, Norwegian University of Science and Technology, Trondheim, Norway.*

[c]*Department of Materials Science and Engineering, Norwegian University of Science and Technology, Trondheim, Norway.*

**Corresponding author: Bjørn Henrik Hansen. E-mail: bjornhenrik.hansen@sintef.no. Phone: +4798283892.*

This SI file contains 9 pages with a total of 6 figures and 2 tables.

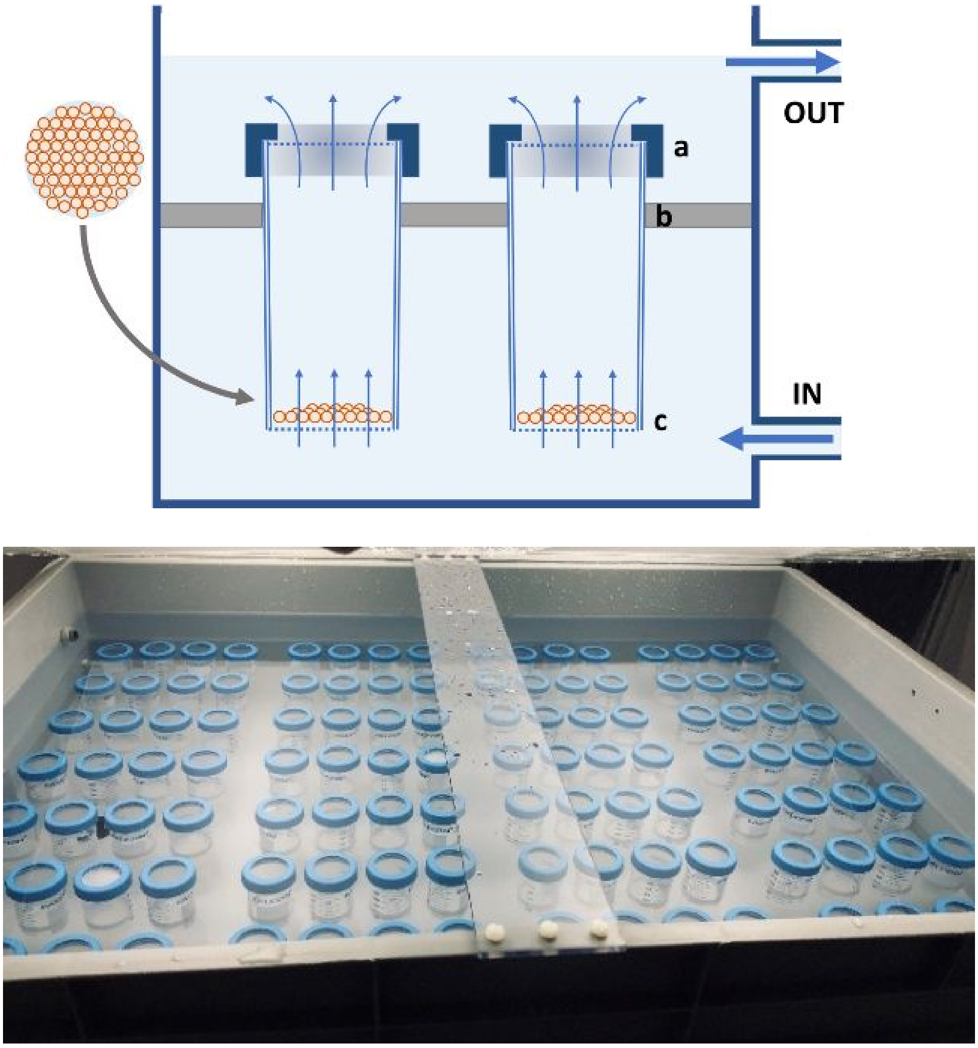

Figure S1. The incubator system. Top: Monolayers of eggs were placed in conical tubes with plankton net in both ends (a, c), and the tubes were fitted into holes of a plate (b) separating the inlet and outlet of a container creating a one-way flow of fresh water through the egg layer. Bottom: Each incubator system consisted of 112 incubator tubes.

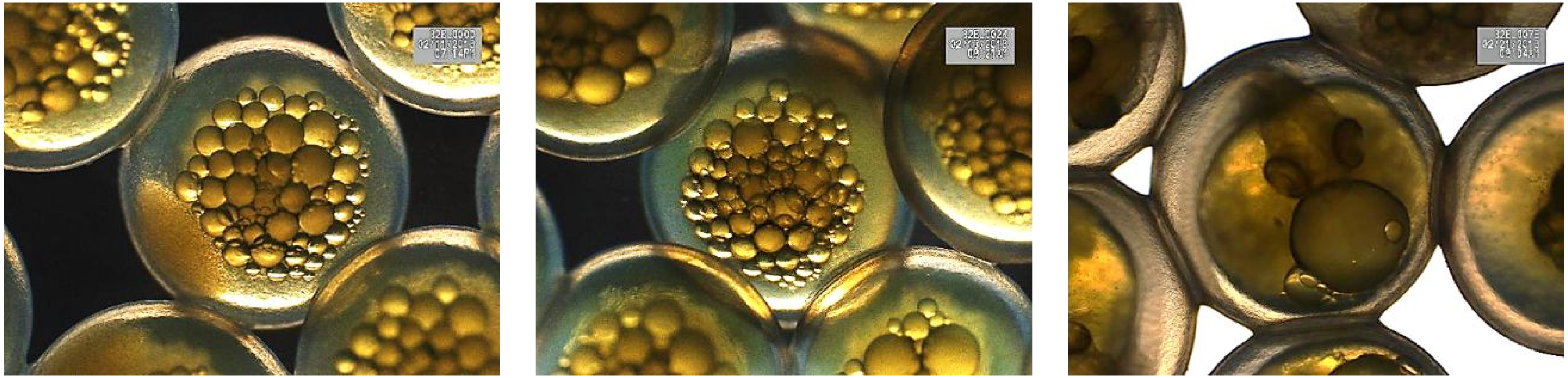

Figure S2: Representative images of embryos taken at the end of the exposure period A: Lumpfish embryos at 48 hpf (DEP1). B: Embryos at 84 hpf (DEP2). C: Embryos at 12 dpf (DEP3).

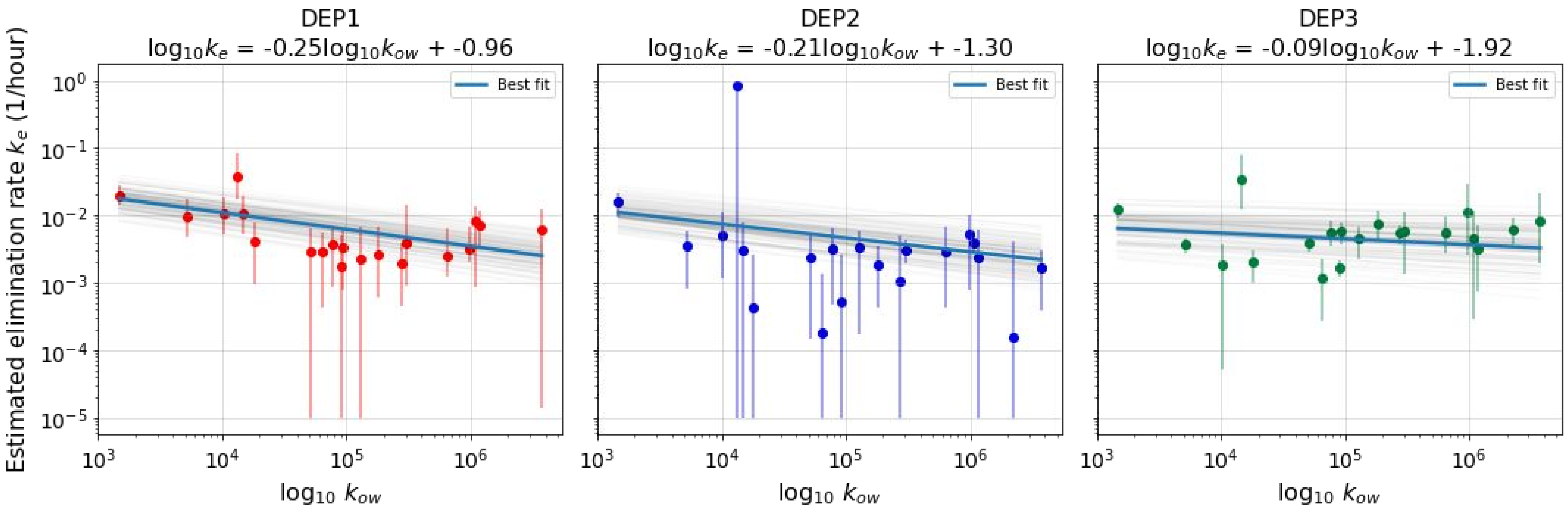

Figure S3. Individually estimated elimination rates of PAHs across k_{ow} values for each DEP calculated using a single-compartment exponential decay model (points and associated error bars). The solid blue line depicts the best fit of the relationship between k_e and k_{ow} (see equation given above each panel). The light grey lines depict the uncertainty of the fit of the regression for each PAH.

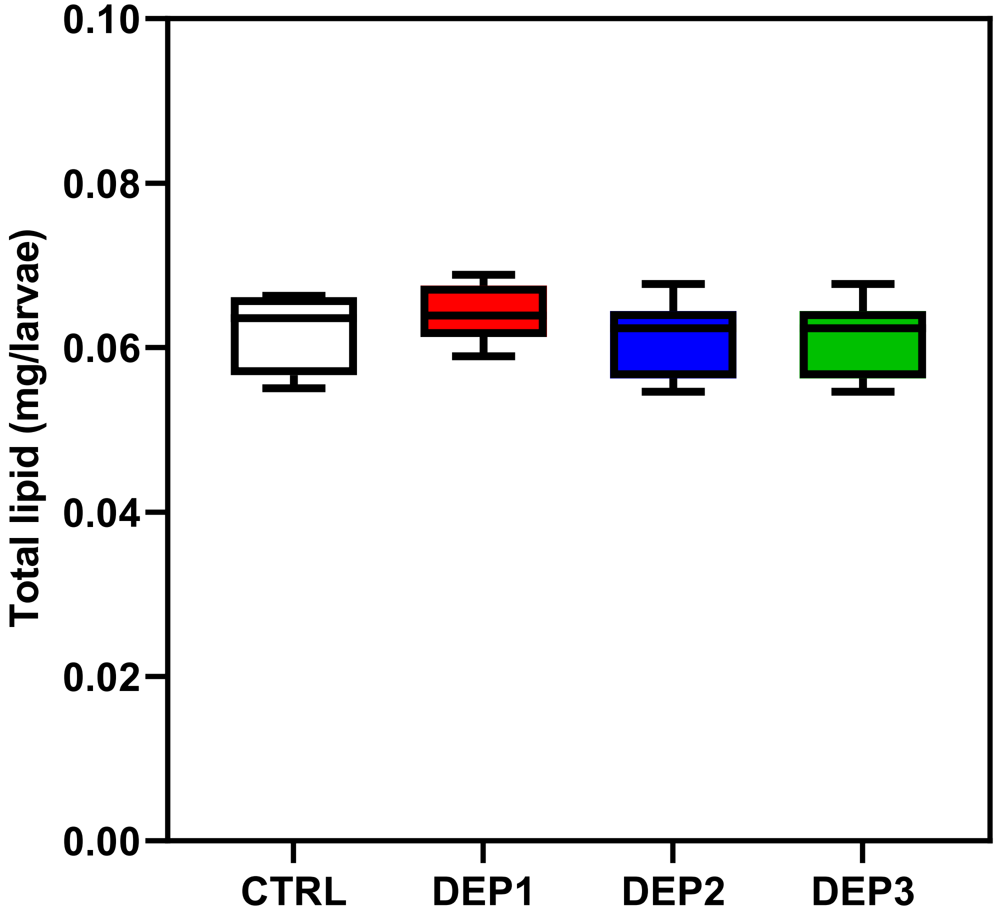

Figure S4: Total lipid content in lumpfish larvae (1 dph) exposed to produced water extract 0-48 hpf (DEP1) 36-84 hpf (DEP2) and 10-12 dpf (DEP3). N=4 for control and N=6 for DEP1-3.

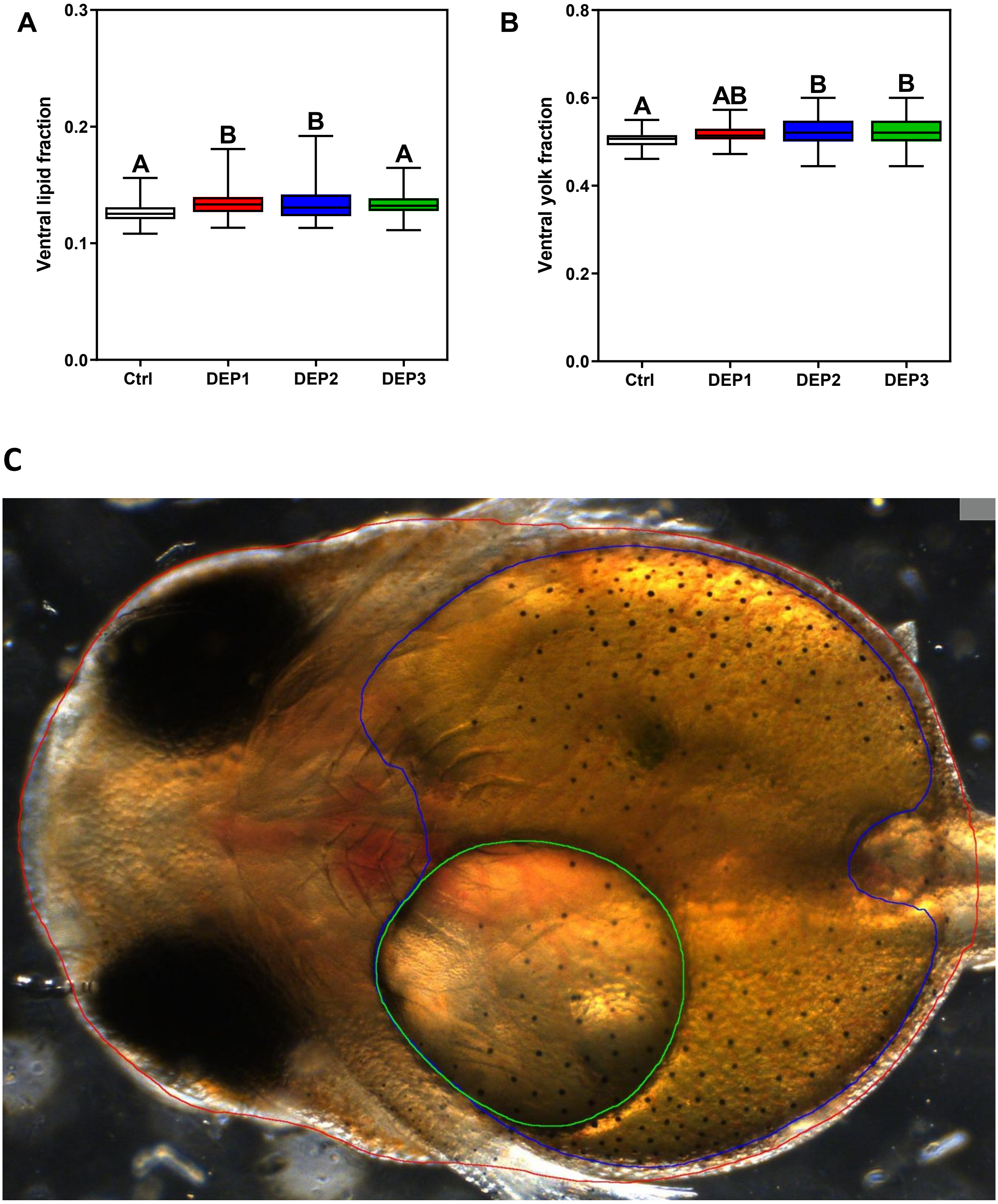

Figure S5. A: Ventral lipid area ((A) and yolk area (B) relative to the body area in lumpfish larvae (1 dph) exposed to produced water extract 0-48 hpf (DEP1) 36-84 hpf (DEP2) and 10-12 dpf (DEP3). N=41, 60, 65 and 65 for control, DEP1, DEP2 and DEP3, respectively. Significant differences between treatment are given with different letters (p<0.05). C: Ventral posture of a lumpfish larvae marked with a body area (red line), yolk area (blue line) and lipid area (green line).

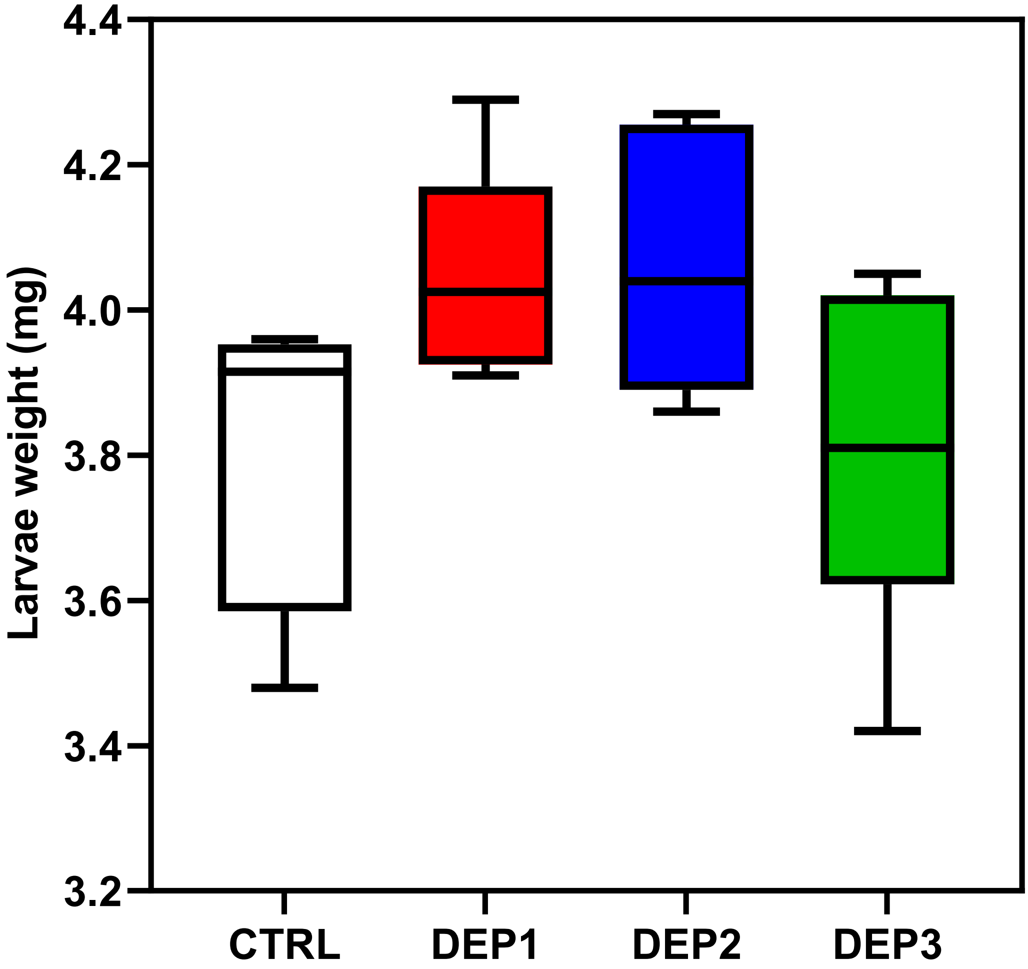

Figure S6: Larvae weight of lumpfish larvae (1 dph) exposed to produced water extract 0-48 hpf (DEP1) 36-84 hpf (DEP2) and 10-12 dpf (DEP3). N=4 for control and N=6 for DEP1-3.

Table S1: Fertilization success (% fertilized eggs), hatching success (% hatched eggs), and main day of hatching (>80% hatch) in days post fertilization (dpf) in lumpfish exposed to produced water extract for 48 h at three different developmental stages of embryogenesis. DEP1 exposed 0-48 hpf. DEP2 exposed 36-84 hpf and DEP3 exposed 10-12 dpf. Controls treated to filtered seawater only. No significant differences were observed between groups (p>0.05)

Treatment	Fertilization (%)	Hatching (%)	Hatch timing (dpf)
**Control**	92.6 ± 3.5 (4)	87.8 ± 6.5 (4)	29 (4)
**DEP1**	92.7 ± 2.0 (6)	90.8 ± 1.8 (6)	30 (6)
**DEP2**	91.4 ± 3.4 (6)	93.4 ± 1.9 (6)	30 (6)
**DEP3**	93.7 ± 1.3 (6)	93.4 ± 1.9 (6)	32 (6)

Table 2: Fatty acid composition (% distribution) of lumpfish larvae (1 dph) exposed to produced water extract 0-48 hpf (DEP1) 36-84 hpf (DEP2) and 10-12 dpf (DEP3). Data are presented as mean ± SD, N=4 for control and N=6 for DEP1-3. Significant differences between treatment are given with different letters (p<0.05).

**Fatty acid**	**Ctrl**	**DEP1**	**DEP2**	**DEP3**
	Mean ± SD (N=4)	Mean ± SD (N=6)	Mean ± SD (N=6)	Mean ± SD (N=6)
C14:0	1.51 ± 0.04	1.48 ±0.03	1.52 ±0.03	1.47 ±0.04
C14:1	0.25 ± 0.12	0.18 ±0.01	0.19 ±0.01	0.18 ±0.00
C15:0	0.35 ± 0.05	0.31 ±0.00	0.32 ±0.01	0.31 ±0.00
C16:0	15.02 ± 0.22	15.01 ±0.28	15.06 ±0.08	14.87 ±0.14
C16:1	2.70 ± 0.03	2.67 ±0.03	2.71 ±0.03	2.67 ±0.04
C17:0	0.74 ± 0.03	0.77 ±0.02	0.78 ±0.01	0.75 ±0.03
C17:1	0.33 ± 0.01	0.32 ±0.01	0.33 ±0.01	0.32 ±0.00
C18:0	4.05 ± 0.06	3.99 ±0.07	3.97 ±0.04	3.98 ±0.05
C18:1n11 +n9	18.06 ± 0.17	18.09 ±0.12	18.10 ±0.17	18.08 ±0.14
C18:1n7	4.07 ± 0.04 (B)	4.02 ±0.07 (B)	4.10 ±0.06 (B)	3.54 ±1.23 (A)
C18:2n6	1.16 ± 0.01	1.16 ±0.01	1.17 ±0.01	1.15 ±0.02
C18:3n6	0.11 ± 0.01	0.13 ±0.01	0.13 ±0.01	0.11 ±0.01
C18:3n3	0.45 ± 0.01	0.46 ±0.01	0.46 ±0.01	0.45 ±0.01
c18:4n3	0.84 ± 0.01	0.85 ±0.01	0.86 ±0.01	0.84 ±0.02
C20:0	0.05 ± 0.01	0.04 ±0.01	0.05 ±0.01	0.04 ±0.00
C20:1	4.49 ± 0.09	4.52 ±0.06	4.47 ±0.05	4.49 ±0.06
C20:2n6	0.18 ± 0.01	0.18 ±0.01	0.19 ±0.01	0.18 ±0.01
c20:3n6	0.11 ± 0.00	0.11 ±0.00	0.11 ±0.00	0.11 ±0.00
C20:4n6	0.62 ± 0.01	0.61 ±0.01	0.62 ±0.01	0.62 ±0.01
C20:3n3	0.07 ± 0.05	0.09 ±0.00	0.09 ±0.00	0.09 ±0.00
c20:4n3	1.01 ± 0.03	1.04 ± 0.01	1.02 ± 0.02	1.02 ± 0.01
C20:5n3	15.73 ± 0.14	15.91 ± 0.08	15.89 ± 0.07	15.93 ±0.04
C22:0	0.03 ± 0.01	0.03 ± 0.00	0.03 ± 0.01	0.03 ±0.00
c22:1n11	0.58 ± 0.02	0.59 ± 0.02	0.57 ± 0.01	0.57 ±0.02
C22:1n9	0.34 ± 0.01	0.35 ± 0.01	0.33 ± 0.00	0.35 ±0.03
C22:2	0.32 ± 0.01	0.32 ± 0.01	0.32 ± 0.01	0.32 ±0.01
C22:3	0.14 ± 0.03	0.16 ± 0.02	0.16 ± 0.03	0.17 ±0.03
C22:4	0.16 ± 0.01	0.16 ± 0.01	0.15 ± 0.00	0.15 ±0.00
c22:5n3	1.25 ± 0.01	1.29 ± 0.03	1.26 ± 0.02	1.29 ±0.02
C24:0	0.00 ± 0.00	0.00 ± 0.00	0.00 ± 0.00	0.00 ±0.00
C22:6n3	19.63 ± 0.17 (A)	20.17 ± 0.37 (B)	19.72 ± 0.25 (A)	20.23 ±0.31 (B)
C24:1n9	0.31 ± 0.02	0.26 ± 0.03	0.24 ± 0.01	0.26 ±0.01
Sum Unassigned peaks	5.40 ± 0.44 (B)	4.76 ± 0.27 (A)	5.14 ± 0.36 (B)	5.44 ±1.23 (B)
Sum sat	21.74 ± 0.22	21.63 ± 0.36	21.72 ± 0.15	21.45 ±0.18
Sum mono	31.10 ± 0.18 (B)	30.99 ± 0.07 (B)	31.03 ± 0.23 (B)	30.46 ±1.35 (A)
Sum poly	41.77 ± 0.34 (A)	42.61 ± 0.48 (B)	42.11 ± 0.29 (A)	42.66 ±0.31 (B)
sum omega 3	38.98 ± 0.33 (A)	39.79 ± 0.48 (B)	39.28 ± 0.30 (A)	39.85 ±0.32 (B)
